# Comorbidity Between Hikikomori and Autistic Traits May Be Identified as a Phenotypical Presentation Characterized by Greater Severity

**DOI:** 10.3390/brainsci15050496

**Published:** 2025-05-10

**Authors:** Liliana Dell’Osso, Benedetta Nardi, Dario Muti, Chiara De Felice, Valeria Tognini, Francesca Parri, Federico Giovannoni, Filippo Del Grande, Chiara Bonelli, Gabriele Massimetti, Stefano Pini, Andrea Fiorillo, Barbara Carpita

**Affiliations:** 1 Department of Clinical and Experimental Medicine, University of Pisa, 56126 Pisa, Italy; liliana.dellosso@unipi.it (L.D.); dario.muti1986@gmail.com (D.M.); c.defelice@studenti.unipi.it (C.D.F.); valeria_tog@hotmail.com (V.T.); francyparri@icloud.com (F.P.); f.giovannoni10@gmail.com (F.G.); chiarabonelli.95@hotmail.it (C.B.); gabriele.massimetti@unipi.it (G.M.); stefano.pini@unipi.it (S.P.); barbara.carpita1986@gmail.com (B.C.); 2 Department of Psychiatry, University of Campania “Luigi Vanvitelli”, 80138 Naples, Italy

**Keywords:** hikikomori, autism, autistic traits, social withdrawal

## Abstract

**Objectives**: Hikikomori is a condition characterized by extreme social withdrawal, functional impairment, and mental distress, which has gained increasing recognition worldwide. While it can be associated with comorbid psychiatric disorders, hikikomori shares similarities with autism spectrum, prompting investigations into their relationship. Given that hikikomori commonly manifests in early adulthood, this study aimed to explore the relationship between autistic features and hikikomori tendencies among university students. **Methods**: A total of 2037 university students were recruited via an online survey and assessed with the Adult Autism Subthreshold (AdAS) Spectrum and the Hikikomori Questionnaire (HQ-25). Participants were categorized into four groups: healthy controls (HCs), subjects with hikikomori tendencies (HKs), subjects with significant autistic traits (ATs), and subjects with both significant ATs and hikikomori tendencies (AT-HKs). **Results**: Results showed significant effects of both hikikomori presence and significant ATs on AdAS Spectrum and HQ-25 scores, while a significant effect of their interaction was detected on AdAS Spectrum scores. The AT-HK group consistently scored higher on both AdAS Spectrum and HQ-25 compared to other groups, with the AT and HK groups outperforming HCs in specific domains. HQ-25 Socialization and Isolation domains predicted higher AdAS Spectrum scores in hikikomori subjects, while various AdAS Spectrum domains served as predictors of HQ-25 scores in AT subjects. **Conclusions**: This study highlights a significant relationship between ATs and hikikomori tendencies in university students, suggesting that their comorbidity may represent a more severe phenotype, where each condition may exacerbate the other.

## 1. Introduction

Hikikomori syndrome is an emerging psychiatric condition characterized by extreme social withdrawal lasting at least six months, accompanied by significant functional impairment or mental distress. The phenomenon was described for the first time in Japan, where it progressively gained interest since the 1990s [1,2,3]. The term “hikikomori” derives from two Japanese characters: “*hiku*” meaning “to pull back” and “*komoru*” meaning “to seclude oneself,” describing an individual “who has withdrawn into seclusion’ [*hikikomotta*], a concept that is deeply intertwined with Japan’s cultural emphasis on community [4]. People with hikikomori typically withdraw from social life, in particular from the workplace or school, for prolonged periods of time, spending extended periods confined to their homes [1,2]. While hikikomori was firstly described as a culture-bound syndrome, its spreading in different countries all over the world has led to a reconceptualization of it as a society-bound syndrome linked to the increasing phenomenon of marginalization in post-industrial society in times of economic crisis [4,5,6,7,8,9,10,11]. In particular, it is possible that individuals with a vulnerability towards psychopathology, including patients affected by other psychiatric disorders, may be more likely to present this specific psychopathological manifestation in specific environmental conditions [10,11].

However, despite its growing recognition, hikikomori is still not yet included in the official diagnostic classification systems, the *Diagnostic and Statistical Manual for Mental Disorder 5th Edition—Text Revision (DSM-5-TR)* and the International Classification of Disease (ICD-11), although described in the section of cultural manifestation of distress of the *DSM-5-TR* [12,13]. Although no formal diagnostic criteria exist, experts agree on certain core features of hikikomori such as severe social isolation, which includes the physical withdrawal in one’s own place of residence, the absence of active participation in academic and labor settings, as well as limited involvement in social relationships, for a duration of at least six months, and the presence of significant functional impairment or distress [9]. Moreover, its prevalence seems to be steadily increasing. Indeed, a survey conducted in Japan in the early 2000s revealed that at least 1.2% of individuals aged 15 to 49 met the criteria for hikikomori [14], while in 2016, the number of affected individuals in this age group had risen to 540,000, with males being disproportionately more impacted—approximately three times more than females [15]. Interestingly, the gender trend appears to reverse in countries outside of Japan, with females being more affected [16].

Moreover, the presence of hikikomori seems to be often associated with other comorbid psychiatric disorders, though research on this is still in its early stages. Common co-occurring disorders include mood disorders, anxiety disorders, psychotic disorders, and personality disorders; however, the relationship between these conditions and prolonged social withdrawal remains under investigation [17]. Furthermore, the potential connection between hikikomori and neurodevelopmental disorders, particularly autism spectrum disorder (ASD), is currently being explored. Indeed, ASD shares strong similarities with hikikomori, with many studies suggesting that individuals with hikikomori may exhibit traits commonly associated with autism spectrum [17,18,19,20].

ASD is a neurodevelopmental disorder characterized by deficits in social communication and interactions, as well as restrictive, repetitive behaviors [13]. The presentation of ASD is highly variable, ranging from individuals with severe intellectual disabilities and limited verbal communication to those with average intelligence, with possibly some field of hyperfunctioning. This broad heterogeneity has led, in recent decades, to a reconceptualization of ASD as a spectrum of traits distributed along a continuum from the general population to the clinical population. In this framework, recent studies have stressed the need to examine not only the full-blown clinical presentations but also the milder, subclinical manifestations of the autism spectrum [21]. Nowadays, it is widely recognized that ASD and even subthreshold autistic traits (ATs), are often associated with other psychiatric comorbidities like anxiety disorders, mood disorders, obsessive-compulsive disorders, and psychosis [22,23,24,25,26,27]. In high-functioning individuals, these comorbidities may mask the underlying neurodevelopmental disorder, as they often compensate for their social difficulties [23,28,29]. In these subjects, stressful life events can trigger the development of psychiatric disorders. In particular, a very delicate phase appears to be that of the transition between high school and university life, which often coincides with the beginning of life as an out-of-town student. In this framework, challenges in social autonomy and living independently may exacerbate a greater difficulty in masking the core difficulties of the disorder, becoming a potential trigger for the development of other psychiatric conditions [30,31,32].

Given these premises and considering that hikikomori commonly begins in adolescence/early adulthood, we sought to investigate the relationship between autistic features and hikikomori, particularly among university students—a group that may be especially vulnerable due to the unique pressures they face. In this context, the aim of our study was to explore the relationship between hikikomori and ATs, focusing on identifying possible specific autistic and hikimomori features presented by subjects with only one or both these conditions.

## 2. Materials and Methods

### 2.1. Study Sample and Procedures

For the aim of this study, we enrolled students of University of Pisa bachelor’s or master’s degrees or of single-cycle degree programs. To encourage participation, all students attending the University of Pisa were invited via email to take part in the study. Participants who agreed to participate provided their consent, completed self-report psychometric assessments, and submitted a form with sociodemographic information. All procedures were conducted anonymously through an online platform.

Students who participated in the study had the option to consult with a psychiatrist to discuss their questionnaire results in more detail. Additionally, no financial compensation or other incentives were offered for participation.

The following self-report questionnaires were used to evaluate each participant: the Adult Autism Subthreshold Spectrum (AdAS Spectrum) for the investigation of ATs and the Hikikomori Questionnaire—25 (HQ—25) for the assessment of hikikomori tendencies.

### 2.2. Measures

#### 2.2.1. Adult Autism Spectrum Questionnaire (AdAS Spectrum)

The AdAS Spectrum is a self-report tool designed to identify both full- and subthreshold ATs and traits in individuals without intellectual disabilities [33]. *Childhood/adolescence*, *verbal* and *non verbal communication*, *empathy*, *inflexibility and adherence to routine*, *restricted interests and rumination*, and *hyper-hypo reactivity to sensory input* are the seven areas that compose the questionnaire’s 160 items. It demonstrates excellent internal consistency, test–retest reliability, and strong convergent validity with other ASD measures [34]. There are two validated threshold scores: a cut-off of 70 for full-blown symptoms of ASD and a cut-off of 43 for clinically significant autistic features [35]. The questionnaire supports a dimensional approach to autism, encompassing threshold-level manifestations, mild/atypical symptoms, gender-specific features, and related personality traits.

#### 2.2.2. Hikikomori Questionnaire—25 (HQ—25)

The HQ-25 is a tool designed to investigate the intensity of hikikomori symptoms experienced over the past six months [36]. It consists of three domains: socialization, emotional support, and isolation. Responses are rated on a 5-point Likert scale from 0 to 4. A validation study established a threshold score of 42 to effectively differentiate individuals at risk for hikikomori from those who are not, demonstrating good sensitivity and specificity. A validated Italian version of the questionnaire is also available [37], demonstrating its cross-cultural applicability. These results affirm the HQ-25 as an effective instrument for screening and studying hikikomori across various cultures and age groups, including adolescents.

### 2.3. Statistical Analysis

The population sample was divided into four groups based on the presence or absence of clinically relevant ATs, of a risk for hikikomori tendencies, or both. The differentiation between subjects with clinically relevant ATs was made based on the threshold score of 43 at the AdAS Spectrum, while the differentiation between subjects at risk of hikikomori and not was made on the basis of the threshold score of 42 in the HQ-25 questionnaire.

Afterwards, chi-square testing and ANOVA were used to compare the sociodemographic variables between the four groups.

Two-way MANOVAs were then performed to assess the effect of the presence/absence of hikikomori and of the presence/absence of significant ATs on the AdAS Spectrum and HQ-25 domains. For this purpose, the AdAS Spectrum and HQ-25 domain scores were used as dependent variables and the presence/absence of hikikomori and of significant ATs, according to HQ-25 and AdAS Spectrum cut-offs, were used as independent variables. Moreover, two factorial ANOVAs were performed to assess the effect of the presence/absence of hikikomori and of the presence/absence of significant AT on the AdAS Spectrum and HQ-25 total scores, with the AdAS Spectrum and HQ-25 total scores used as dependent variables and the presence/absence of hikikomori and of significant ATs, according to HQ-25 and AdAS Spectrum cut-offs, as independent variables.

A one-way ANOVA analysis followed by post hoc Bonferroni corrections were also conducted in order to compare the scores obtained on the AdAS Spectrum and HQ-25 questionnaires among the four different groups and in order to confirm the results from the MANOVA and factorial ANOVAs.

Then, we performed linear regression to investigate which HQ-25 domains were predictive of the presence of higher AdAS Spectrum scores in the hikikomori group. For this purpose, we used the presence of clinically relevant ATs as dependent variable and the HQ-25 domains as independent variables.

Lastly, a second linear regression analysis was conducted to investigate which AdAS Spectrum domains were predictive of hikikomori tendencies in the AT group. For this purpose, we used HQ-25 as the dependent variable and AdAS Spectrum domains as independent variables.

All statistical analyses were performed with SPSS version 26.0.

## 3. Results

The total sample consisted of 2037 students enrolled in three-year, master’s, or single-cycle degree programs. Participants were divided into four groups based on the presence or absence of clinically relevant ATs, of a risk for hikikomori tendencies, or both:

- A total of 550 subjects without clinically relevant ATs and at no risk of hikikomori (HCs);
- A total of 118 subjects with significant hikikomori tendencies but without significant ATs (HKs);
- A total of 818 subjects with significant ATs but without hikikomori (ATs);
- A total of 821 subjects who had significant ATs and also showed hikikomori tendencies (AT-HKs).

The four groups significantly differed in gender and age (see Table 1), with the AT group being significantly younger. Females were more represented in the HC group, while males predominated in the AT-HK group.

**Figure 1:**
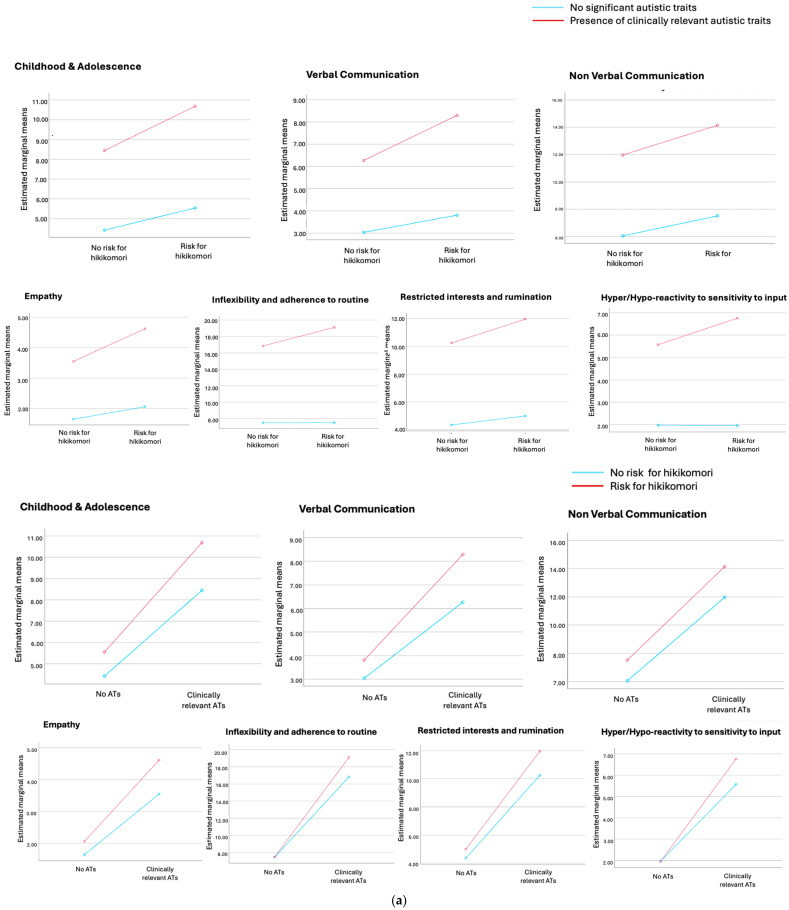

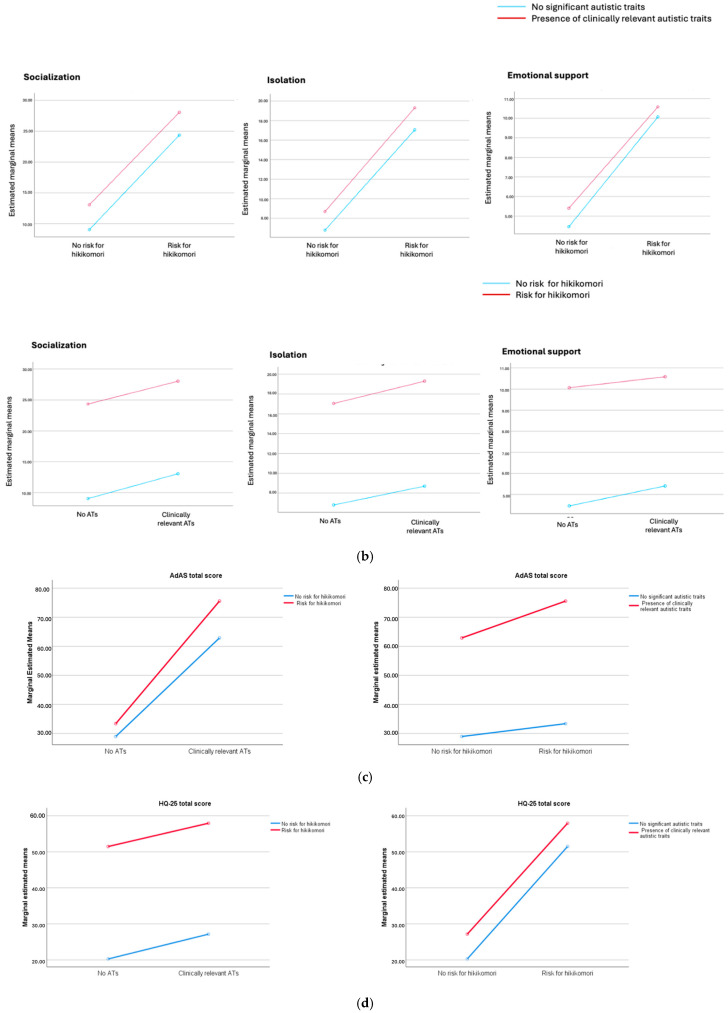
(**a**): Profile plot of estimated marginal means for AdAS domains depending on presence of hikikomori and ATs. (**b**): Profile plot of estimated marginal means for HQ-25 domains depending on presence of hikikomori and AT. (**c**): Profile plot of estimated marginal means for AdAS Spectrum total score depending on presence of hikikomori and Ats. (**d**): Profile plot of estimated marginal means for HQ-25 total score depending on presence of hikikomori and Ats.

MANOVA results using AdAS Spectrum domains as dependent variables showed a significant effect of both hikikomori presence (Wilks’ lambda = 0.932, F = 24.112, *p* < 0.001) and significant ATs (Wilks’ lambda = 0.563, F = 256.142, *p* < 0.001), as well as their interaction (Wilks’ lambda = 0.989, F = 3.791, *p* < 0.001) on all AdAS Spectrum domains —except nonverbal communication (Table 2a, Figure 1a). Regarding HQ-25 domains, significant effects were found for hikikomori (Wilks’ lambda = 0.453, F = 924.828, *p* < 0.001) and ATs presence (Wilks’ lambda = 0.944, F = 45.398, *p* < 0.001), but no significant interaction was observed (Wilks’ lambda = 0.999, F = 0.834, *p* = 0.475) (Table 2a, Figure 1b). Two-factorial ANOVAs confirmed significant effects of both hikikomori and ATs on AdAS total score (AT: F = 1713.334, *p* < 0.001; hikikomori: F = 86.511, *p* < 0.001) and HQ-25 total score (AT: F = 128.484, *p* < 0.001; hikikomori: F = 2774.002, *p* < 0.001). A significant effect of hikikomori and ATs interaction was observed for AdAS total scores (F = 20.212, *p* < 0.001) but not for HQ-25 total scores (F = 0.128, *p* = 0.721) (Table 2b,c; Figure 1c,d).

**Figure 2:**
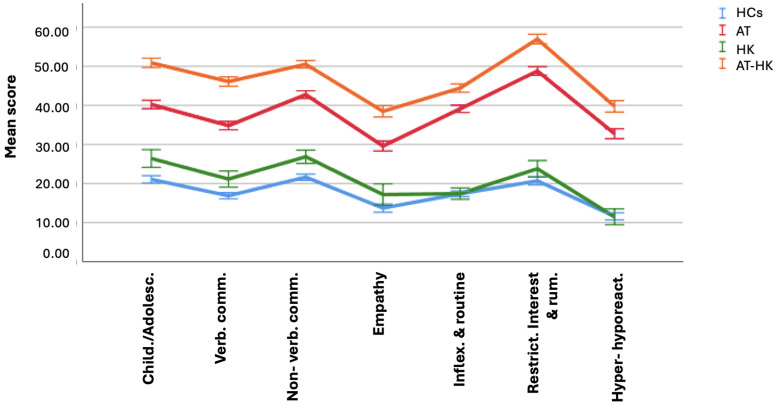
Comparison of AdAS Spectrum domains score among groups.

**Figure 3:**
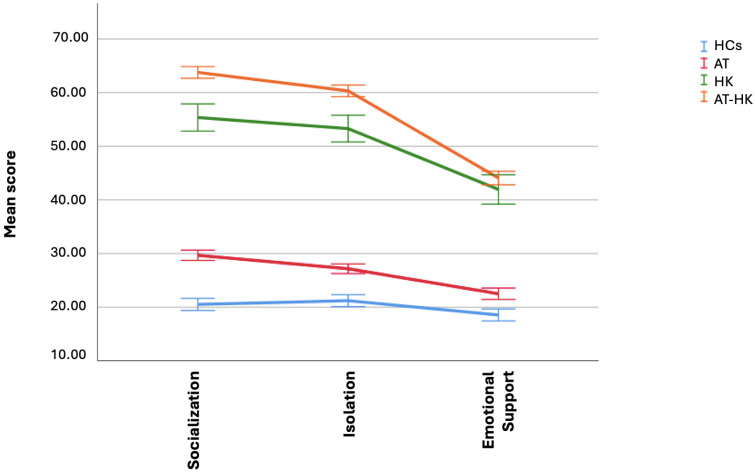
Comparison of HQ-25 domains’ score among groups.

ANOVA results (Table 3, Figure 2 and Figure 3) show that the AT-HK group scored significantly higher than all other groups on AdAS total and in multiple domains: *childhood/adolescence*, *verbal communication*, *empathy*, *inflexibility and adherence to routine*, *rumination and restricted interests*, and *hyper-hyporeactivity to sensory input.* The AT group scored higher than both HK and HC groups, which did not differ significantly. For the *nonverbal communication* domain, scores followed a descending order: AT-HK > AT > HK > HC. For the HQ-25, AT-HK scored highest on total score and the *socialization* and *isolation* domains, followed by HK, then AT, with HCs lowest. In the *emotional support* domain, AT-HK and HK groups scored higher than AT, which outperformed HCs.

A Linear regression in the HK group (Table 4) showed that the HQ-25 *socialization* and *isolation* domains significantly predicted higher AdAS scores. Another regression in the AT group identified AdAS domains—*childhood/adolescence*, *verbal* and *non verbal communication*, *empathy*, and *restricted interests and rumination*—as significant predictors of higher HQ-25 scores. Interestingly, *inflexibility and adherence to routine* emerged as a negative predictor (Table 5).

## 4. Discussion

This study examined the link between ATs and hikikomori tendencies among university students, focusing on how these conditions manifest individually and together. Findings revealed that both hikikomori risk and clinically relevant ATs significantly affected all domains of the AdAS Spectrum, both independently and interactively. Specifically, while higher ATs naturally influence AdAS Spectrum scores, the presence of hikikomori also has a significant impact on these scores, highlighting the complex and interconnected relationship between the two conditions. The interaction between ATs and hikikomori on AdAS Spectrum scores suggests that among individuals with elevated ATs, those who also experience hikikomori tend to show a notably greater increase in autism severity compared to AT subjects without hikikomori. In contrast, within the nonautistic population, the presence of hikikomori is associated with only a modest increase in autism-related symptoms. This indicates that the effect of hikikomori on autism severity is much more pronounced in individuals with high ATs than in those without.

Our results also showed a significant effect of both hikikomori risk and clinically relevant ATs on all domains of the HQ-25: in particular, not only subjects with hikikomori tendencies showed, as expected, higher hikikomori symptoms, but also subjects with ATs, suggesting that subjects with ATs would be at higher risk to show greater hikikomori symptoms. These results align with previous research suggesting that, due to core challenges in social communication, ATs may predispose individuals to develop hikikomori [20,38,39,40]. Indeed, many authors have proposed that challenges in social interactions and in communication proper of autism may lead to social withdrawal, increasing vulnerability towards hikikomori tendencies, up to its extreme pathological drift [41]. Emphasizing the importance of neurodevelopmental alterations, some studies have suggested that ASD and hikikomori may share a neurodevelopmental basis. In this framework, the innate propensity for seclusion exhibited by individuals with high levels of ATs—referred to by some authors as “hikikomori affinity”—and the ability to occasionally predict the full-blown onset of hikikomori syndrome lend credence to this notion [20,38,39].

Earlier studies, particularly during the COVID-19 pandemic, have emphasized the link between ATs and heightened social withdrawal [42]. A recent systematic review examining the effects of quarantine on individuals with ASD reported that, after the significant reduction in social interactions caused by confinement measures, ASD individuals experienced stronger behavioral effects from isolation, including anxiety, irritability, and emotional dysregulation [42]. Similarly, neurodevelopmentally atypical children facing loneliness show mental health risks with likely long-term effects [43]. In our data, individuals with both ATs and hikikomori scored higher on all AdAS Spectrum and HQ-25 measures, suggesting that individuals with comorbid ATs and hikikomori represent a distinct phenotype marked by greater severity in both conditions. This supports the notion that ATs may increase susceptibility to severe hikikomori, while hikikomori may be more likely in those with elevated ATs. Globally, these findings provide additional support for the hypotheses previously proposed in the existing literature, suggesting that while the presence of ATs may generally increase the vulnerability towards higher hikikomori tendencies, eventually playing the role of a predisposing factor, among subjects in the autism spectrum, hikikomori tendencies would more likely be specifically associated with more pronounced ATs, as evidenced also by the interaction between the two conditions on autism spectrum severity. Interestingly, the group with only hikikomori was much smaller than the comorbid group (12.6% vs. 87.4%), while ATs were evenly distributed between those with and without hikikomori (50.01% vs 49.99%). Additionally, while AT-only individuals had elevated HQ-25 scores, hikikomori-only individuals did not differ significantly from controls on most AdAS items, except for nonverbal communication. These findings suggest that most hikikomori cases involve co-occurring ATs, while those with ATs, although more constantly presenting a higher tendency towards non-extreme social isolation, may also present with different psychopathological manifestations with respect to hikikomori. In this framework, neuroatypicality may act as a broader vulnerability factor, shaping diverse psychopathological outcomes through interactions with environmental and biological influences. The specific timing, nature, and severity of neurodevelopmental alterations—combined with interactions between biological and environmental factors—may shape the trajectory of illness, contributing to the emergence of diverse clinical presentations and comorbidities, including, in this context, hikikomori [31,41]. On the other hand, it could be hypothesized an eventual role of other psychopathological dimensions in the limited number of subjects with hikikomori without significant ATs, such as mood disorders, psychosis, or trauma- or stress-related disorders, which would be in line with the acknowledged presence of hikikomori secondary to other mental conditions [3,31,41].

Even in the absence of significant ATs, individuals with hikikomori exhibited marked deficits in nonverbal communication. This finding is particularly significant given that nonverbal communication accounts for approximately 65% of all human communication and plays a key role in conveying emotional content [44]. This aligns with studies showing that hikikomori individuals experience reduced social competence and communication, which may contribute to their gradual withdrawal [38]. Early signs, like difficulty making friends, being bullied, or preferring solitude, have also been linked to hikikomori onset. Further supporting this hypothesis, research has shown that changes in social interaction and communication difficulties, such as difficulty in joining a group of friends or making new ones, a preference for solitary activities, and being bullied, are not only early warning signs of a tendency towards social isolation but also significant predictors of the subsequent development of hikikomori [45,46,47].

Our results also suggest that stronger difficulties in the HQ-25 domains of *socialization* and *isolation* predict clinically relevant ATs in hikikomori subjects. Difficulties in social interaction and communication are central to ASD diagnosis [13] and may stem from deficits in social motivation and neurobiological functioning—especially in brain regions tied to social reward processing, such as the orbitofrontal–striatal–amygdala network [48,49,50,51]. Dysregulation of oxytocin, a key social bonding hormone, may further impair social motivation in ASD [52]. Many individuals with high ATs demonstrate a longstanding preference for isolation, likely influenced by both social impairments and the effort required to cope in social environments [41]. This can lead to avoidance and, in some cases, escalate to full-blown hikikomori [53]. Difficulties with group inclusion, a preference for solitary activities, and restricted interests may all contribute to this process [20].

Furthermore, several AdAS Spectrum domains—including *childhood/adolescence*, *verbal* and *non verbal communication*, *empathy*, and *restricted interests*—were predictive of more severe hikikomori symptoms among AT individuals. Indeed, not only are social challenges in childhood—such as struggles with joining peer groups, making new friends, a preference for solitary activities, and experiences of bullying—key predictors of the subsequent development of hikikomori [45,46,47] but deficits in both verbal and nonverbal communication have also been identified as contributing factors to the gradual process of social withdrawal [38]. Similarly, narrow and intensely pursued interests such as Internet and video game use have frequently been documented in hikikomori, both of which may act as a predisposing factor leading to social withdrawal [54,55].

Interestingly, the AdAS Spectrum domain of *inflexibility and adherence to routine* emerged instead as a negative predictor of the risk for hikikomori. This evidence, although apparently counterintuitive, may reflect how autistic rigidity, while limiting flexibility, can provide a protective structure that discourages total withdrawal by reinforcing daily routines [40].

### Limits

Important limitations should be considered when evaluating these results. Only university students were included in the sample, so the results cannot be extrapolated to the wider population. Moreover, participants were selected voluntarily, and potential biases in sample selection, such as an over-representation of participants with a greater interest in the topic, should be considered. Thirdly, the fact that we relied on self-reported questionnaires raises the possibility of over- or underestimation of symptoms. Finally, the cross-sectional nature of the study means that we cannot infer causal or temporal relationships between the variables studied.

## 5. Conclusions

In conclusion, this study provides evidence of a significant relationship between ATs and hikikomori tendencies among university students. Our findings highlight how subjects with comorbid ATs and hikikomori should be identified as a phenotype characterized by a greater severity of both conditions, supporting the hypothesis that ATs may be a vulnerability factor for developing more severe forms of hikikomori while, on the other hand, hikikomori conditions would be more likely underlain by greater ATs. Moreover, our study also suggests the presence of specific patterns of features that may help in profiling subjects with both hikikomori and ATs: in particular, while a deeper isolation and socialization difficulties may be indicative of the underlying presence of ATs in hikikomori subjects, the presence of greater ATs since childhood, communication and empathy impairments, as well as a greater tendency towards ruminative thinking may be more likely associated with the development of hikikomori in AT subjects. While these results contribute to a deeper understanding of the association between hikikomori and ATs, additional research, especially longitudinal studies, is warranted to better investigate temporal relationships and to apply these findings to different populations.

## Figures and Tables

**Table 1:** Age and gender comparison between groups.

		HCs Mean ± SD n = 550	HK Mean ± SD n = 118	AT Mean ± SD n = 818	AT-HK Mean ± SD n = 821	F	*p*
**Age**		25.23 ± 6.35	26.08 ± 9.28	23.87 ± 5.25	24.76 ± 5.85	8.344	<0.001 *
		**n(%)**	**n(%)**	**n(%)**	**n(%)**	**Chi-square**	***p***
**Gender**	**F**	316 ^a^ (57.5%)	61 ^b^ (51.7%)	449 ^b^ (54.9%)	410 ^a^ (49.9%)	8.428	0.038 *
	**M**	234 ^b^ (42.5%)	57 ^b^ (48.3%)	369 ^b^ (45.1%)	411 ^b^ (50.1%)		

* Significant for *p* < 0.05; * HCs, HK, AT-HK > AT. Each superscript letter indicates a subset of categories in which the row proportions are not different from each other.

**Table 2:** (**a**) Results from the two MANOVAs with the AdAS Spectrum domains and HQ-25 domains as dependent variables and presence of significant ATs and hikikomori as independent variables. (**b**) Factorial ANOVA with AdAS Spectrum total score as dependent variables and presence of significant ATs and hikikomori as independent variables. (**c**) Factorial ANOVA with HQ-25 total score as dependent variables and presence of significant ATs and hikikomori as independent variables.

(**a**)						
**Source**	**Dependent Variable**	**Type III Sum of Squares**	**df**	**Mean Square**	**F**	***p***
**Corrected Model**	**AdAS Spectrum**					
	*Child./adolesc.*	13,812.181	3	4604.060	445.208	<0.001 *
	*Verb. comm.*	9756.571	3	3252.190	451.629	<0.001 *
	*Non verb. comm.*	23,747.788	3	7915.929	606.636	<0.001 *
	*Empathy*	3137.983	3	1045.994	217.743	<0.001 *
	*Inflex. and routine*	54,413.152	3	18,137.717	566.395	<0.001 *
	*Restrict. interest and rum.*	22,224.705	3	7408.235	672.611	<0.001 *
	*Hyper-hyporeact.*	8942.784	3	2980.928	326.227	<0.001 *
	**HQ-25**					
	*Socialization*	152,004.066	3	50,668.022	1234.124	<0.001 *
	*Isolation*	70,214.727	3	23,404.909	1134.953	<0.001 *
	*Emotional support*	17,129.499	3	5709.833	381.433	<0.001 *
**Intercept**	**AdAS Spectrum**					
	*Child./adolesc.*	66,469.476	1	66,469.476	6427.534	<0.001 *
	*Verb. comm.*	35,970.629	1	35,970.629	4995.214	<0.001 *
	*Non verb. comm.*	123,677.907	1	123,677.907	9478.033	<0.001 *
	*Empathy*	11,065.245	1	11,065.245	2302.440	<0.001 *
	*Inflex. and routine*	203,188.264	1	203,188.264	6345.060	<0.001 *
	*Restrict. interest and rum.*	78,145.272	1	78,145.272	7094.997	<0.001 *
	*Hyper-hyporeact.*	20,717.238	1	20,717.238	2267.254	<0.001 *
	**HQ-25**					
	*Socialization*	435,825.126	1	435,825.126	10,615.419	<0.001 *
	*Isolation*	211,012.747	1	211,012.747	10,232.446	<0.001 *
	*Emotional support*	73,062.762	1	73,062.762	4880.799	<0.001 *
**Hikikomori**	**AdAS Spectrum**					
	*Child./adolesc.*	888.591	1	888.591	85.926	<0.001 *
	*Verb. comm.*	613.847	1	613.847	85.244	<0.001 *
	*Non verb. comm.*	1037.606	1	1037.606	79.517	<0.001 *
	*Empathy*	172.322	1	172.322	35.872	<0.001 *
	*Inflex. and routine*	414.861	1	414.861	12.955	<0.001 *
	*Restrict. interest and rum.*	439.493	1	439.493	39.903	<0.001 *
	*Hyper-hyporeact.*	107.811	1	107.811	11.799	0.001 *
	**HQ-25**					
	*Socialization*	72,216.835	1	72,216.835	1758.990	<0.001 *
	*Isolation*	34,208.393	1	34,208.393	1658.836	<0.001 *
	*Emotional support*	9145.719	1	9145.719	610.960	<0.001 *
**Significant ATs**	**AdAS Spectrum**					
	*Child./adolesc.*	6605.892	1	6605.892	638.783	<0.001 *
	*Verb. comm.*	4678.825	1	4678.825	649.745	<0.001 *
	*Non verb. comm.*	12,358.944	1	12,358.944	947.125	<0.001 *
	*Empathy*	1562.486	1	1562.486	325.261	<0.001 *
	*Inflex. and routine*	34,552.256	1	34,552.256	1078.980	<0.001 *
	*Restrict. interest and rum.*	13,016.314	1	13,016.314	1181.782	<0.001 *
	*Hyper-hyporeact.*	5548.491	1	5548.491	607.16	<0.001 *
	**HQ-25**					
	*Socialization*	4686.351	1	4686.351	114.146	<0.001 *
	*Isolation*	1356.006	1	1356.006	65.756	<0.001 *
	*Emotional support*	167.459	1	167.459	11.187	0.001 *
Hikikomori * Significant ATs	**AdAS Spectrum**					
	*Child./adolesc.*	97.697	1	97.697	9.447	0.002 *
	*Verb. comm.*	124.349	1	124.349	17.268	<0.001 *
	*Non verb. comm.*	39.189	1	39.189	3.003	0.083
	*Empathy*	33.545	1	33.545	6.983	0.008 *
	*Inflex. and routine*	399.672	1	399.672	12.481	<0.001 *
	*Restrict. interest and rum.*	89.889	1	89.889	8.161	0.004 *
	*Hyper-hyporeact.*	115.274	1	115.274	12.615	<0.001 *
	**HQ-25**					
	*Socialization*	8.526	1	8.526	0.208	0.649
	*Isolation*	9.465	1	9.465	0.459	0.498
	*Emotional support*	15.115	1	15.115	1.010	0.315
**Error**	**AdAS Spectrum**					
	*Child./adolesc.*	23,816.163	2303	10.341		
	*Verb. comm.*	16,583.948	2303	7.201		
	*Non verb. comm.*	30,051.617	2303	13.049		
	*Empathy*	11,063.134	2303	4.804		
	*Inflex. and routine*	73,749.114	2303	32.023		
	*Restrict. interest and rum.*	25,365.558	2303	11.014		
	*Hyper-hyporeact.*	21,043.870	2303	9.138		
	**HQ-25**					
	*Socialization*	94,551.640	2303	41.056		
	*Isolation*	47,492.298	2303	20.622		
	*Emotional support*	34,474.587	2303	14.969		
**Total**	**AdAS Spectrum**					
	*Child./adolesc.*	190,278.000	2307			
	*Verb. comm.*	111,956.000	2307			
	*Non verb. comm.*	338,318.000	2307			
	*Empathy*	40,851.000	2307			
	*Inflex. and routine*	641,911.000	2307			
	*Restrict. interest and rum.*	242,057.000	2307			
	*Hyper-hyporeact.*	86,458.000	2307			
	**HQ-25**					
	*Socialization*	994,960.000	2307			
	*Isolation*	474,846.000	2307			
	*Emotional support*	173,087.000	2307			
**Corrected total**	**AdAS Spectrum**					
	*Child./adolesc.*	37,628.344	2306			
	*Verb. comm.*	26,340.518	2306			
	*Non verb. comm.*	53,799.404	2306			
	*Empathy*	14,201.117	2306			
	*Inflex. and routine*	128,162.266	2306			
	*Restrict. interest and rum.*	47,590.264	2306			
	*Hyper-hyporeact.*	29,986.654	2306			
	**HQ-25**					
	*Socialization*	246,555.707	2306			
	*Isolation*	117,707.025	2306			
	*Emotional support*	51,604.087	2306			
(**b**)						
**Source**	**Type III Sum of Squares**		**df**	**Mean Square**	**F**	***p***
**Corrected model**	808,948.507		3	269,649.502	1014.480	0.000 *
**Intercept**	3,163,381.852		1	3,163,381.852	11,901.334	0.000 *
**Significant AT**	455,405.354		1	455,405.354	1713.334	0.000 *
**Hikikomori**	22,994.576		1	22,994.576	86.511	0.000 *
Significant AT * Hikikomori	5372.392		1	5372.392	20.212	0.000 *
**Error**	612,138.823		2303	265.801		
**Total**	9,123,597.000		2307			
**Corrected total**	1,421,087.330		2306			
(**c**)						
**Source**	**Type III Sum of Squares**		**dF**	**Mean Square**	**F**	***p***
**Corrected model**	616,619.289		3	205,539.763	1889.519	0.000 *
**Intercept**	1,931,635.792		1	1,931,635.792	17,757.456	0.000 *
**Significant AT**	13,976.323		1	13,976.323	128.484	0.000 *
**Hikikomori**	301,752.755		1	301,752.755	2774.002	0.000 *
adascut43 * HQcut	13.921		1	13.921	0.128	0.721
**Error**	250,517.709		2303	108.779		
**Total**	4,147,795.000		2307			
**Corrected total**	867,136.999		2306			

(**a**) *: Statistically significant value (*p* < 0.05); df: degrees of freedom; (**b**) *: statistically significant value (*p* < 0.05); df: degrees of freedom; R^2^ = 0.569; adjusted R^2^ = 0.569. (**c**) *: statistically significant value (*p* < 0.05); df: degrees of freedom; R^2^ = 0.711; adjusted R^2^ = −0.711.

**Table 3:** Comparison of AdAS Spectrum and HQ-25 scores among groups.

	HCs Mean ± SD n = 550	HK Mean ± SD n = 118	AT Mean ± SD n = 818	AT-HK Mean ± SD n = 821	F	*p*
**AdAS Spectrum scores**						
**Child./adolesc.**	4.42 ± 2.41	5.54 ± 2.61	8.45 ± 3.29	10.68 ± 3.66	445.208	<0.001 *
**Verb. comm.**	3.04 ± 1.74	3.80 ± 2.04	6.27 ± 2.78	8.29 ± 3.14	451.629	<0.001 *
**Non verb. comm.**	6.05 ± 2.66	7.52 ± 2.60	11.97 ± 3.98	14.14 ± 3.89	606.636	<0.001 °
**Empathy**	1.64 ± 1.52	2.06 ± 1.80	3.55 ± 2.25	4.62 ± 2.54	217.743	<0.001 *
**Inflex. and routine**	7.46 ± 3.72	7.48 ± 3.43	16.82 ± 5.81	19.10 ± 6.73	566.395	<0.001 *
**Restrict. interest and rum.**	4.34 ± 2.61	4.99 ± 2.43	10.24 ± 3.32	11.96 ± 3.81	672.611	<0.001 *
**Hyper-hyporeact.**	1.97 ± 1.84	1.95 ± 1.89	5.57 ± 3.09	6.76 ± 3.65	326.277	<0.001 *
**Total score**	28.93 ± 9.45	33.35 ± 7.03	62.87 ± 16.46	75.56 ± 20.24	1014.480	<0.001 *
**HQ-25**						
**Socialization**	9.03 ± 5.91	24.35 ± 6.11	13.06 ± 6.13	28.05 ± 7.01	1234.124	<0.001 ^
**Isolation**	6.79 ± 4.22	17.05 ± 4.38	8.69 ± 4.17	19.30 ± 5.09	1134.953	<0.001 ^
**Emotional support**	4.45 ± 3.21	10.07 ± 3.60	5.40 ± 3.69	10.58 ± 4.44	381.433	<0.001 ^§^
**Total score**	20.27 ± 10.26	51.47 ± 8.66	27.15 ± 9.58	57.93 ± 11.53	1889.519	<0.001 ^

* AT-HK > AT > HK, HCs; ° AT-HK > AT > HK > HCs; ^ AT-HK > HK > AT > HCs; ^§^: AT-HK, HK > AT > HCs; significant for *p* < 0.05.

**Table 4:** Linear regression analysis with the presence of higher AdAS Spectrum scores as a dependent variable and HQ-25 domains as independent variables, carried in the HK group.

	B (S.E)	BETA	t	*p*
*Constant*	27.405 (3.645)		7.519	<0.001 *
**Socialization**	1.090 (0.112)	0.323	9.748	<0.001 *
**Isolation**	0.547 (0.158)	0.117	3.451	0.001 *
**Emotional support**	0.227 (0.169)	0.042	1.341	0.180

* Significant for *p* < 0.05; R^2^ = 0.151; Adjusted R^2^ = 0.148.

**Table 5:** Linear regression analysis with higher HQ-25 scores as a dependent variable and AdAS Spectrum domains as independent variables, carried in the AT group.

	B (S.E.)	BETA	t	*p*
*constant*	11.968 (1.618)		7.399	<0.001 *
**Child./adolesc.**	1.044 (0.132)	0.204	7.913	<0.001 *
**Verb. comm.**	1.247 (0.170)	0.209	7.339	<0.001 *
**Non verb. comm.**	0.617 (0.123)	0.135	5.030	<0.001 *
**Empathy**	0.792 (0.185)	0.104	4.280	<0.001 *
**Inflex. and routine**	−0.249 (0.089)	−0.085	−2.787	0.005
**Restrict. interest and rum.**	0.481 (0.151)	0.095	3.194	0.001
**Hyper-hyporeact.**	−0.104 (0.151)	−0.019	−0.686	0.493

* Significant for *p* < 0.05; R^2^ = 0.242; Adjusted R^2^ = 0.239.

## Data Availability

All data generated or analyzed during this study are included in this published article.
